# Comparison of amiodarone and esmolol for prevention of reperfusion ventricular fibrillation in individuals undergoing heart valve or aortic surgery: a study protocol for a randomized controlled clinical trial

**DOI:** 10.1186/s13063-023-07816-w

**Published:** 2023-11-27

**Authors:** Dan Zhu, Yu Li, A-yong Tian, Hong-nan Wang

**Affiliations:** Department of Anesthesiology, The First Hospital of China Medical University, 155 Nanjing North Street, Heping District, Shenyang City, Liaoning Province China

**Keywords:** Amiodarone, Esmolol, Reperfusion ventricular fibrillation, Blood drainage volume

## Abstract

**Background:**

Amiodarone and esmolol can help to prevent and treat post-cardiac surgery reperfusion ventricular fibrillation. However, the relative efficacies of these two drugs remain unknown. The aim of the current trial is to compare the performances of amiodarone and esmolol for preventing reperfusion ventricular fibrillation following open heart surgery.

**Methods/design:**

This is a single-center, prospective, double-blind, controlled clinical trial. A total of 260 patients undergoing heart valve or aortic surgery will be assigned randomly to treatment with prophylactic esmolol (intervention group) or amiodarone (control group). The main outcome is the incidence of reperfusion ventricular fibrillation following aortic opening during extracorporeal circulation. The secondary outcomes are the rate of automatic cardiac resuscitation, energy and frequency of electrical defibrillation, number of electrical defibrillations, and pacemaker use in the two groups of patients. Information on the patients’ general condition and the durations of anesthesia, extracorporeal circulation, aortic occlusion, and operation time will be recorded. We will also compare the heart rate, mean arterial pressure, and central venous pressure between the two groups of patients at induction of anesthesia (T1), start of surgery (T2), start of extracorporeal circulation (T3), aortic block (T4), aortic opening (T5), after opening for 10 (T6), 20 (T7), and 30 min (T8), at cessation of extracorporeal circulation (T9), and at the end of surgery (T10) and compare blood gas analysis results at T1, T5, T9, and T10.

**Discussion:**

This study will determine if prophylactic esmolol is more effective than amiodarone for reducing the incidence of reperfusion ventricular fibrillation in patients undergoing heart valve or aortic surgery.

**Trial registration:**

China Clinical Trials Registry ChiCTR1900026429. Registered on 2019.10.9.

## Background

Advancements in surgical methods, aids, and cardiopulmonary bypass (CPB) techniques have increased the indications for open heart surgery. However, cardiac surgery and CPB are still associated with potentially fatal consequences, such as bleeding, infection, and arrhythmias. Arrhythmias are the most severe complication, and ventricular fibrillation (VF) is the most severe secondary arrhythmia [1, 2], with reperfusion VF occurring in 70–96% of cases of CPB [3, 4]. Pathophysiologically, VF is associated with aortic opening-induced myocardial ischemia and reperfusion injury, together with the ensuing inflammatory reactions, oxidative stress, and electrical instability [5]. Electrical defibrillation has been the gold standard treatment for VF after aortic opening; however, electrical defibrillation may aggravate myocardial damage and lead to cardiac dysfunction, as well as recurrent and fatal arrhythmias [6]. Preventive measures are therefore needed to reduce the risk of VF after aortic opening during open heart surgery and improve patient outcomes.

Antiarrhythmic medications have been shown to be effective for treating VF following aortic opening during cardiac surgery, with amiodarone and esmolol chosen as the representative medicines for clinical use. As a class III antiarrhythmic medication, the main electrophysiological mechanism of amiodarone involves lengthening the action potential and effective refractory period of cardiac tissue, hence preventing return-excitation. Amiodarone also slows the conduction rate by inhibiting the fast influx of sodium ions into the conduction fibers of the atria and myocardium. However, its ability to prevent VF is inconsistent [7, 8]. Esmolol is a selective beta 1 blocker with a very short half-life, which has been shown to accelerate spontaneous cardiac recovery following aortic opening and decrease the incidence of reperfusion VF [9].

Their pharmacological properties and past research suggest that both amiodarone and esmolol can successfully prevent and cure reperfusion VF following CPB. We hypothesized that esmolol would be more effective in this regard, and accordingly designed a clinical trial to compare the efficacies of amiodarone and esmolol for preventing reperfusion VF during open heart surgery.

## Methods/design

### Study design {8}

This is a prospective single-center, double-blind, superiority randomized controlled clinical trial to compare the efficacies of amiodarone and esmolol for preventing reperfusion VF during open heart surgery in patients undergoing heart valve or aortic surgery.

Amiodarone has been shown to increase survival, improve hemodynamic parameter maintenance, and reduce myocardial damage in an experiment by Zoerner et al. during direct cardiac surgery [10]. Exogenous epinephrine has long been acknowledged as a crucial medication for cardiac resuscitation despite the significant release of endogenous catecholamines during VF. Post-defibrillation dysfunction and arrhythmias are more likely to happen when the heart’s metabolic needs have increased and its energy reserves are depleted. Exogenous epinephrine also affects cardiac control by altering the activity of the cardiac nervous system. Esmolol, an ultrashort-acting beta-adrenergic receptor blocker, has been demonstrated to drastically lower hypertension, tachycardia, and arrhythmias brought on by intubation and surgical stimulation when administered as a preventative measure in patients having coronary artery surgery. The effects of these two commonly used drugs have rarely been compared.

## Study site and patient selection

### Patients

A total of 260 patients who underwent heart valve or aortic surgery at the Department of Cardiac Surgery, The First Affiliated Hospital of China Medical University, Shenyang, Liaoning province, China, starting November 1, 2019, are recruited. The purpose and potential risks and benefits of the study are fully explained to the patients and their families before surgery, and informed consent will be obtained.

### Inclusion and exclusion criteria

The inclusion criteria are ASA class II to III and a signed informed consent form. The exclusion criteria are an allergy to amiodarone or esmolol, history of bronchial asthma, history of thoracotomy, history of malignant arrhythmia, and abnormal liver, kidney, or thyroid function.

### Who will take informed consent? {26a}

Informed consent is obtained from the anesthetist during the pre-operative visit or from the blinded members of the study team on the day of surgery. Written informed consent is also required from the patient’s legal representative and the investigator before any study-related procedures are started (26a).

### Additional consent provisions for collection and use of participant data and biological specimens {26b}

Not applicable, collection of biological samples is not required.

### Recruitment {15}

In addition to being examined at the preoperative visit, any patient getting ready for heart surgery must schedule an anesthetic evaluation exam at our facility’s anesthesia clinic. The anesthesiologist will describe the trial and respond to any queries from the parents or legal representative during the session.

The researcher will thoroughly explain to participants the research questions, the advantages of the protocol, and the positive impact of the inquiry on public health in order to encourage them to actively participate throughout the study. Additionally, subjects will be urged to be accessible afterwards to call the investigator for counseling and medical guidance based on trial-related biochemical signs. Free medical services will be provided at each visit enquiry, while these services are not required.

## Interventions

### General anesthesia

All patients will receive conventional general anesthesia as follows: induction with midazolam 0.05 mg/kg, etomidate 0.3 mg/kg, sufentanil 0.5 µg/kg, and cis-atracurium 0.2 mg/kg. The anesthesia machine is connected after tracheal intubation, the tidal volume is set at 6–8 mL/kg, and the respiratory rate is 12 breaths/min. The intraoperative end-tidal CO_2_ pressure is maintained at 35–45 mmHg by adjusting the respiratory parameters, and the bispectral index (BIS) is maintained at 40–60. Anesthesia will be maintained with continuous pumping of propofol 4–8 mg/kg/h, with scheduled replenishment of sufentanil and cis-atracurium. Heparin 300 IU/kg is given intravenously at the beginning of the operation, and esmolol is administered to patients in the intervention group after the activated clotting time reached > 480 s. Esmolol will be administered by a single-dose bolus (usually 500 μg/kg) followed by a continuous infusion (between 100 and 300 μg/kg/min or titrated in order to maintain low heart rates). Patients in the control group will receive extracorporeal circulation and the surgical operation is completed, and 300 mg of amiodarone is then administered 10 min before aortic opening. Drug infusion is stopped 10 min after opening the aorta in both groups. If electrical defibrillation is required after aortic opening, defibrillation is initially performed using 20 J, and if this is ineffective twice, defibrillation is repeated using 30 J. If defibrillation is unsuccessful after 3 attempts, a second dose of study medication will be administered on the basis of prior randomization. Patients in the control group will receive an additional 150 mg of amiodarone. At the end of the procedure, the patient will be returned to the cardiac surgery unit under ventilatory ball-assisted ventilation.

A five-lead electrocardiogram, ambulatory blood pressure, oxygen saturation, end-expiratory CO_2_ pressure, BIS, and nasopharyngeal temperature will be monitored throughout the procedure and blood gas analysis will be performed at regular intervals.

### Strategies to improve adherence to interventions {11c}

To encourage better study compliance, the researcher will thoroughly explain the trial to each patient and give them instructions for taking both medications. If any concerns or health problems arise while they are subjects in the study, the researcher will get in touch with them. Since the focus of this pilot project is perioperative indicators, it is unique that the investigator will carry out the majority of the experimental procedures, ensuring a particular level of adherence.

### Relevant concomitant care permitted or prohibited during the trial {11d}

Not applicable. If it is a necessary therapeutic intervention to preserve the patient’s life, it will not be discontinued. Groups that do not fit the trial’s primary study criteria may be excluded.

### Outcome {12}

#### Primary outcome measure

The main outcome is the incidence of VF following aortic opening within 30 min during extracorporeal circulation.

#### Secondary outcome measures

The secondary outcomes are the energy and frequency of electrical defibrillation, pacemaker use, anesthesia time, CPB time, aortic occlusion time, and operation time in the two groups.

**Fig. 1:**
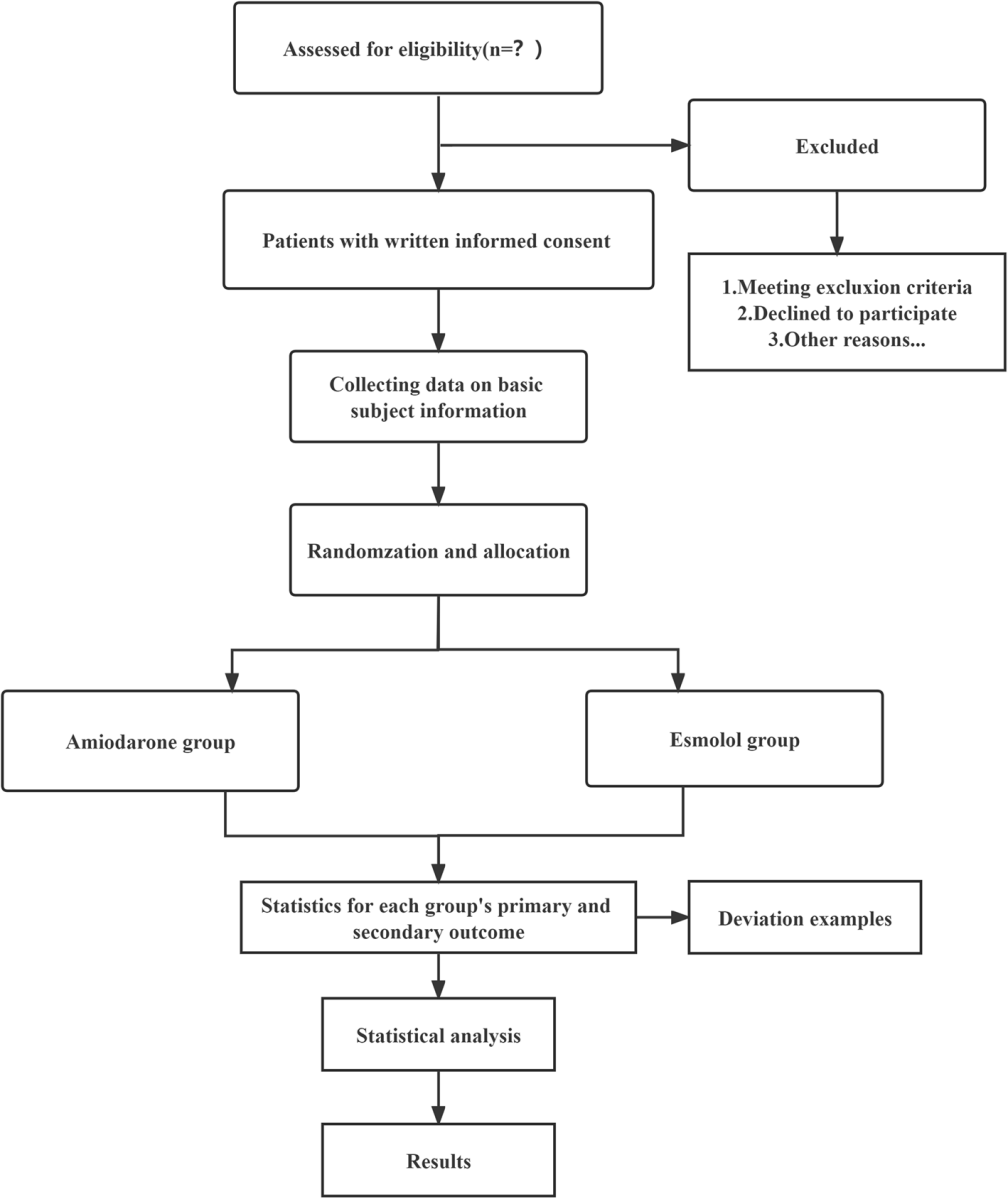
Flowchart

The other data are heart rate, mean arterial pressure (MAP), and central venous pressure (CVP) at the time of anesthesia induction (T1), surgery (T2), cardiopulmonary bypass (T3), aortic occlusion (T4), aortic opening (T5), after opening for 10 (T6), 20 (T7), and 30 min (T8), at cessation of cardiopulmonary bypass (T9), and completion of surgery (T10). Blood gas analysis results at T1, T5, T9, and T10 are also recorded and compared (Fig. 1).

### Participant timeline {13}

**Table:** 

*Event*	*Timepoint*					
	**Preoperative**		**Intraoperative**			
	***T*0**	***T1T2T3***	***T4***	***T5***	***T6T7T8***	***T9T10***
**Recruitment**						
Eligibility screening	**X**					
Informed consent	**X**					
Random allocation	**X**					
**Intervention**						
Esmolol			**X**			
Amiodarone			**X**			
**Data collation**						
Base variable	**X**	**X**	**X**	**X**	**X**	**X**
Primary outcomes					**X**	
Secondary outcomes				**X**	**X**	**X**
Other data	**X**	**X**	**X**	**X**	**X**	**X**
**Assesment**						
Incidence of ventricular fibrillation					**X**	

*T0* pre-entry, *T1 *anesthesia induction, *T2 *surgery begins, *T3 *cardiopulmonary bypass, *T4 *aortic occlusion, *T5 *aortic opening, *T6 *after opening for 10 min, *T7 *20 min, *T8 * 30 min, *T9 *at cessation of cardiopulmonary bypass, *T10 *completion of surgery

### Determination of sample size

We determined the required sample size based on the prevalence of VF as the primary outcome. A previous retrospective study found that the incidences of VF are 39% [11] and 23% [12] in patients treated with amiodarone or esmolol, respectively, prior to aortic opening. A sample size of 127 patients per group is appropriate to finish the trial to detect a difference with 80% power and a 5% type I error rate. Given the potential for patient dropouts, a sample of 130 patients per group is required.

## Assignment of interventions: allocation

### Implementation {16b}

Following randomization, information will be placed in envelopes that are sealed. A nurse anesthetist from the study group will open the envelopes on the day of surgery in the order of assignment, and esmolol and amiodarone will be provided based on the grouping. An anesthesiologist who is not informed of the grouping will then conduct the study. The outcome evaluation will be carried out by a trained anesthesiologist, and the data analysis will be done by a different anesthesiologist. They are not both aware of the group assignment. At the preoperative visit, the patient signs an informed consent form, which is then given to the principle investigator.

### Enrolling {16c}

After seeing the patient, the anesthesiologist is in charge of recruiting participants.

### Double-blind randomization and allocation concealment

The 260 patients will be divided randomly into the intervention group and control group in a ratio of 1:1 using a computer-generated sequence.

### Blinding {17a}

The anesthesiologist will not be informed of the patient assignments, which are recorded and sealed in sequentially numbered opaque envelopes. Nurse anesthetists can give sealed envelopes and tell them to prepare the items for the experiment based on group allocations; there are no differences in the look of the two sets of envelopes. The group assignment will be withheld from all patients and anesthesiologists. Following anesthesia induction, the anesthesiologist may perform the various surgeries in accordance with the coded number. Another anesthesiologist will document intraoperative data and follow up on the patient’s discharge. Finally, the data will be analyzed by a professional statistician.

### Unblinding {17b}

A blinding committee will be convened, which consisted of experts to oversee and guide the blinding process. At the end of the trial, after the completion of the locking library, in order to carry out the analysis, the first level of blinding, so as to know each subject as group A or B, but do not know the specific group A and B group operation. On the basis of primary blinding, statistical analysis is performed to clarify the difference between group A and group B. Secondary blinding will be then performed to know the operation group information of each subject, to clarify whether it is the intervention group or the control group, and to further analyze the results of the clinical trial. The blinding results will be recorded in a blinding form and confirmed and signed under the supervision of a blinding committee. Under the direction of a blinding committee, the outcomes of the blinding will be documented in a blinding form and verified and signed.

The data will not be released until the final statistical analysis has been completed.

## Data collection and management

### Plans for assessment and collection of outcomes {18a}

Dedicated staff will record all data and complete a case report form for each patient. Relevant demographic characteristics and the patients’ past medical histories, including age, height, weight, and diagnosis, will be collected at the time of patient inclusion. Intraoperative data including heart rate, MAP, and CVP will be recorded at the induction of anesthesia (T1), start of surgery (T2), start of extracorporeal circulation (T3), aortic block (T4), aortic opening (T5), after opening for 10 (T6), 20 min (T7), and 30 min (T8), at cessation of extracorporeal circulation (T9), and at the end of surgery (T10), and blood gas analysis will be recorded at T1, T5, T9, and T10.

### Plans to promote participant retention and complete follow-up {18b}

Not applicable. We believe that the focus of our experiment is on various markers during the perioperative phase. On the day of the procedure, the patient will be monitored until the patient is discharged from the PACU. We shall employ the same data collecting and statistical forms for all experimental data.

### Data management {19}

The Principal Investigator is accountable for the data’s correctness, completeness, readability, and timeliness. Investigators will keep correct medical history report forms and background material for research participants in their medical records.

### Confidentiality {27}

All researchers are professional clinical or scientific experts and will strictly abide by the regulations on patient information protection and ethical safety. Personal information that has been recorded is handled with the utmost confidentiality and processed and stored in line with data protection legislation. Except for parties directly involved in the subject’s care and organizations to whom the subject has given express agreement, no patient-identifiable information will be transmitted to anyone else.

### Statistical analysis

Statistical analysis will be performed using SPSS software, version 26.0 for Windows (SPSS, Inc., Chicago, IL, USA). Continuous variables will be described as mean (standard deviation) or median (25% and 75% percentile) and will be analyzed using independent *t*-tests or Mann–Whitney *U*-tests, respectively. Categorical variables will be described as a frequency or percentage and will be analyzed by *χ*^2^ tests. Prior to statistical analysis, each continuous variable will be analyzed to determine if it is normally distributed. If the assumption of a normal distribution is violated, the appropriate transformation will be used.

### Subgroup analyses {20b}

At this moment, subgroup analyses have not been taken into account. The variables employed in the minimization algorithm will be subjected to subgroup analyses, but only for the main result, if necessary.

### The treatment of non-adherence and missing data {20c}

Analyses will be performed with intention-to-treat: all participants will be analyzed according to the block to which they are randomly assigned. In addition, the trial includes the intraoperative administration of two drugs and the monitoring of intraoperative-related data. The amount of data lost is expected to be minimal.

The researchers will make every effort to collect all follow-up data on all participants, and almost all data will be collected in the perioperative period. Therefore, it is expected that missing data will be minimal. Subjects with missing co-primary outcome data will not be included in the primary analysis in the first place. Each randomized group’s result data is summarized separately. By comparing baseline characteristics of subjects with missing and imputed values, potential bias owing to missing data will be examined. Missing predictors can be identified using the number of missing values. The missing predictors will subsequently be included as covariates in the main analytical model via a sensitivity analysis. Various multiple interpolation algorithms can also be used if necessary.

### Interim analyses {21b}

Regular meetings will be held to evaluate and correct the trial as necessary. However, at this trial, interim analyses are not planned.

## Oversight and monitoring

### Experimental steering and data monitoring committee {5d}

A team of researchers will manage or supervise this single-center study. To monitor study progress and track study status, the primary investigator will hold monthly phase meetings (overall study and study design), as well as a study steering committee meeting to maintain ongoing contact between investigators and discuss study progress. A core team of the principal investigator, an experienced cardiac surgeon, a clinical pharmacist, and a data statistician are in charge of the study’s conduct, adherence to the study protocol, patient safety, and the review of new information related to the study questions. The trial will not require a data monitoring committee because the risk of adverse outcomes is low. Of course, any major adverse events, problems, or injuries will be reported to and documented by the CMU Scientific Ethics Committee.

### Reported adverse events {22}

Any negative experience a participant has while taking part in the study is referred to as an adverse event (AE), regardless of whether it is thought to be related to the intervention. From the moment of drug application to day 3 following surgery, AEs will be carefully observed in this trial. The investigator will keep track of all AE information, including type, diagnosis date, duration, care, and outcomes. The Principal Investigator will be informed of any serious adverse events and will judge the severity and causality of these events. All authorized entities associated with the study will be recorded and reported to the Ethics Committee as part of the annual report.

The study will be stopped if there is a serious adverse event (SAE).SAEs include death, disability, and severe allergic reactions that seriously affect the health of the patient.

### Plans for communicating important protocol modification {25}

If problems are discovered and the trial protocol needs to be modified or adjusted, the reasons, contents, and timing of the modification should be stated, and the application should be submitted to the Ethics Committee for approval and consent before implementation; if the modification of the trial protocol during the clinical trial involves the informed consent of the subjects, the informed consent form should be changed; or if there is a new modification or addition to the trial protocol, the informed consent form should be changed; or if there is a new modification or addition.

## Discussion

Continuous improvements in surgical techniques and the prudent use of anti-inflammatory drugs have greatly reduced the incidence of complications such as bleeding and infection after direct cardiac surgery, causing reperfusion VF after CPB aortic opening to become a more prominent and fatal complication. The mechanism of reperfusion VF is complex and it is currently thought to be caused by a combination of factors resulting in decreased and increased non-conductivity of myocardial cells during the non-reperfusion period. Reperfusion VF induces a severe stress response characterized by increased sympathetic activity, myocardial oxygen consumption, myocardial ischemia, and myocardial injury.

Intraoperative hypothermia and cold myocardial protective fluid are currently used to reduce the occurrence of reperfusion VF, but it remains a potentially fatal complication after myocardial reperfusion.

Antiarrhythmic drugs are commonly used by cardiac anesthesiologists to prevent and treat VF. Previous research showed that amiodarone not only improved the metabolic efficiency of the heart after ischemia reperfusion, but also reduced the transmural dispersion of cardiac repolarization, which is strongly linked to the development of reperfusion VF [13, 14]. In contrast, esmolol has been proposed to enhance the overall balance of myocardial oxygen supply and demand during resuscitation, potentially by minimizing the delay in cardiomyocyte depolarization produced by receptor excitation, thereby lowering the heart’s oxygen demand [15]. However, high-quality human study data are lacking.

The results of the current study may be useful in clinical settings, to lessen myocardial damage and thereby enhance survival. We reduced the impact of differences between individual anesthesiologists and cardiac surgeons by analyzing surgical procedures performed by the same surgical team, with all anesthesia provided by a single anesthesiologist.

The drug doses and administration techniques used in this study are chosen based on prior studies; however, the doses of amiodarone and esmolol used may not be sufficient to achieve therapeutic concentrations, taking into account the increased circulating volume of the CPB circuit.

The aim of this study is to determine whether amiodarone or esmolol is more effective for preventing the onset of reperfusion VF and enhancing the pace of automatic cardiac resumption after aortic opening. However, the causes of reperfusion-induced VF are complex, and we anticipate the need for further studies to address this question.

## Study status

Patient recruitment begins in November 2019. The expected study completion date is March 2024.

## Disclosure policy

In accordance with conventional procedures, the trial’s unblinded data will not be available until the main results have been released. A clinical article describing the study’s primary and secondary outcomes will be published, regardless of the magnitude or nature of the impact, and a complete report, anonymized participant-level dataset, and the statistical codes used to produce the results will be made publically available no later than 3 years following the study’s conclusion. The authors received no industry backing for this study.

## Patient and public involvement

Patients and/or the public are not involved in the design, conduct, reporting, or dissemination plans of this research.

## Data Availability

Data and materials are available on reasonable request. Additional data may be made available upon request.
